# Isolation of subtelomeric sequences of porcine chromosomes for translocation screening reveals errors in the pig genome assembly

**DOI:** 10.1111/age.12548

**Published:** 2017-05-12

**Authors:** R. E. O'Connor, G. Fonseka, R. Frodsham, A. L. Archibald, M. Lawrie, G. A. Walling, D. K. Griffin

**Affiliations:** ^1^ School of Biosciences University of Kent Canterbury CT2 7AF UK; ^2^ Cytocell Ltd Newmarket Road Cambridge UK; ^3^ The Roslin Institute R(D)SVS University of Edinburgh Division of Genetics and Genomics Easter Bush Midlothian EH25 9RG UK; ^4^ JSR Genetics Southburn Driffield North Humberside YO25 9ED UK

**Keywords:** bacterial artificial chromosome, food production, hypoprolificacy, karyotype

## Abstract

Balanced chromosomal aberrations have been shown to affect fertility in most species studied, often leading to hypoprolificacy (reduced litter size) in domestic animals such as pigs. With an increasing emphasis in modern food production on the use of a small population of high quality males for artificial insemination, the potential economic and environmental costs of hypoprolific boars, bulls, rams etc. are considerable. There is therefore a need for novel tools to facilitate rapid, cost‐effective chromosome translocation screening. This has previously been achieved by standard karyotype analysis; however, this approach relies on a significant level of expertise and is limited in its ability to identify subtle, cryptic translocations. To address this problem, we developed a novel device and protocol for translocation screening using subtelomeric probes and fluorescence *in situ* hybridisation. Probes were designed using BACs (bacterial artificial chromosomes) from the subtelomeric region of the short (p‐arm) and long (q‐arm) of each porcine chromosome. They were directly labelled with FITC or Texas Red (p‐arm and q‐arm respectively) prior to application of a ‘Multiprobe’ device, thereby enabling simultaneous detection of each individual porcine chromosome on a single slide. Initial experiments designed to isolate BACs in subtelomeric regions led to the discovery of a series of incorrectly mapped regions in the porcine genome assembly (from a total of 82 BACs, only 45 BACs mapped correctly). Our work therefore highlights the importance of accurate physical mapping of newly sequenced genomes. The system herein described allows for robust and comprehensive analysis of the porcine karyotype, an adjunct to classical cytogenetics that provides a valuable tool to expedite efficient, cost effective food production.

## Introduction

The domestic pig (*Sus scrofa domesticus*) provides 43% of meat consumed worldwide, making it the leading source of meat protein globally (US Department of Agriculture 2015). Purebred boars selected for their genetic merit are used at the top (nucleus) level of the breeding pyramid, meaning that any fertility problems in these animals could significantly reduce litter sizes throughout the breeding population. This ultimately leads to a reduction in food production and higher environmental costs per mating animal, issues that are perpetuated further through an increasing emphasis on artificial insemination (AI) (Kahn & Line 2010).

Semen used in AI preparations is routinely assessed for parameters that are considered to be indicative of fertility such as sperm concentration, morphology and motility. Evidence suggests that these parameters are, in fact, not reliable indicators of prolificacy (Gadea 2005). Indeed, the primary identification of boars that exhibit hypoprolificacy is deduced from both litter sizes and ‘non‐return rates’, i.e. the proportion of sows/gilts served by that boar that return to heat (i.e. fail to conceive) after 21 days. With a gestation length of 114 ± 2 days and an average born alive litter size of 12 piglets, each sow can produce around 23 slaughter pigs per year, assuming there are no fertility problems (BPEX 2014). In addition, fertility is assessed using farrowing rates, which indicate how many litters are produced against how many sows were originally served (ideally >85%) (Gadea *et al*. 2004). The mating of hypoprolific boars into the sow population can have a significant effect on non‐return rates and litter sizes, in some cases reducing the number of piglets in a litter by up to 50%. In order to prevent the perpetuation of reduced fertility, the identification and elimination of hypoprolific boars from the breeding population is a priority, particularly given rising global populations and increasing demand for meat products, with per capita consumption of pig meat expected to reach 15.1 kg/year by 2030 (Bruinsma 2003).

Balanced chromosomal rearrangements occur frequently in pigs and are seen in as many as 0.47% of AI boars awaiting service (Ducos *et al*. 2007). Over 130 reciprocal translocations have been identified, with chromosomes 1, 7, 14 and 15 the most frequently involved (Rothschild & Ruvinsky 2011). Reciprocal translocations adversely affect reproductive performance in pigs by causing a reduction in litter size due to high mortality among early embryos. Approximately 50% of boars exhibiting hypoprolificacy are reciprocal translocation carriers, even though they have a normal phenotype and semen parameters (Rodríguez *et al*. 2010). Balanced translocations are considered to be the primary reason for hypoprolificacy in pigs due to the generation of unbalanced gametes and subsequent partially aneuploid conceptuses that lead to early loss of zygotes and ultimately litters that are 25–50% smaller than would be expected (Gustavsson 1990; Pinton *et al*. 2000).

Since the latter part of the 20th century, several continental European programmes of chromosomal screening have been established, with the largest centre of pig screening being based at the National Veterinary School of Toulouse, France (Ducos *et al*. 2008). This has led to the identification of a significant number of chromosomal rearrangements in otherwise phenotypically normal boars. However, since this period, there has been a reduction in the number of laboratories that perform animal cytogenetics (with approximately 10–15 operating worldwide, mostly in Europe) (Ducos *et al*. 2008).

Current translocation screening is performed by Giemsa‐banding (G‐banding) and routine karyotyping. Although this is simple and cost effective, it requires specialist knowledge of the porcine karyotype and is limited in its ability to detect translocations smaller than 2–3 Mb in size, especially if bands of similar intensity are exchanged. Moreover, even in the best laboratories, preparations of sub‐optimal quality (e.g. yielding few preparations, which are difficult to analyse) can occasionally arise. Such is the nature of biological systems, and in these cases, molecular cytogenetics can aid detection protocols. The recent sequencing of the pig genome provided the tools through which molecular cytogenetic resources can be identified and developed for more accurate and unequivocal translocation screening. Results from our own laboratory provided evidence that the strategy of assembling the swine genome clone‐by‐clone ahead of whole genome sequencing provided the ability to select a clone for fluorescence *in situ* hybridisation (FISH) with 100% confidence that it would map to the predicted chromosomal position. That is, of 71 clones selected, all mapped to the predicted chromosome band (Groenen *et al*. 2012).

In humans, Knigh *et al*. (1997) demonstrated an approach through which cryptic (sub‐microscopic) translocations could be identified in humans using a FISH strategy that involved 24 individual hybridisations (one for each chromosome) on a single slide. By hybridising to the subtelomeric regions of the short (p) and long (q) arms of each chromosome, each in a different colour, any chromosome translocation is clearly visible, even to the untrained eye. This approach has been used extensively in clinical cytogenetics (Horsley *et al*. 1998; Dawson *et al*. 2002; Ravnan *et al*. 2006) and, to some degree, in pigs (Mompart *et al*. 2013). The purpose of the current study was to develop these investigations further to generate a panel of equivalent porcine FISH probes, extending the study by Knight *et al*. to develop a porcine version of the human system. The aim was to employ a strategy that would significantly increase the speed and accuracy of boar translocation screening, the ultimate objective being the identification and removal of hypoprolific boars from the breeding population. This could potentially improve efficiency as well as reduce the cost and environmental footprint of global meat production.

## Materials and methods

### Chromosome preparations

In order to generate the material for screening and for the identification of potential translocation carriers, we established a routine karyotyping service for UK companies wishing to screen their boars for translocations. Blood samples were provided by three of the UK's leading pig breeding companies (JSR Genetics, ACMC and Genus PIC). Heparinized blood samples were cultured for 72 h in PB MAX Karyotyping medium (Invitrogen) at 37 °C, 5% CO_2_. Cell division was arrested by adding colcemid at a concentration of 10.0 μg/ml (Gibco) for 35 min before hypotonic treatment with 75 m KCl and fixation to glass slides using 3:1 methanol:acetic acid. Metaphases for karyotyping were stained with DAPI in VECTASHIELD^®^ antifade medium (Vector Laboratories). Image capturing was performed using an Olympus BX61 epifluorescence microscope with a cooled CCD camera and SmartCapture (Digital Scientific UK) system. smarttype software (Digital Scientific UK) was used for karyotyping purposes after being custom‐adapted for porcine karyotyping according to the standard karyotype as established by the Committee for the Standardized Karyotype of the Domestic Pig (Gustavsson 1988). All staff were trained in the analysis of porcine chromosomes using the in‐house developed program karyolab porc (Payne *et al*. 2009).

### Selection and preparation of subtelomeric bacterial artificial chromosome clones for fluorescence *in situ* hybridisation

Bacterial artificial chromosome (BAC) clones of approximately 150 kb in size were selected using the Sscrofa Version 10.2 NCBI database (www.ncbi.nlm.nih.gov) for each autosome and the X chromosome. A lack of available BACs for the Y chromosome meant that this chromosome was excluded from the study. End‐sequenced BACs in the subtelomeric region of the p‐arm and q‐arm of each chromosome with unique placement in the genome were identified and ordered from both the PigE‐BAC library (Anderson *et al*. 2000) and the CHORI‐242 Porcine BAC library (BACPAC). BAC DNA was isolated using the Qiagen Miniprep Kit, the products of which were then amplified and directly labelled by nick translation with FITC‐Fluroescein‐12‐UTP (Roche) for p‐arm probes and Texas Red‐12‐dUTP (Invitrogen) for q‐arm probes prior to purification.

### Development of a novel Multiprobe device for translocation screening

Fluorescently labelled probes were diluted to a concentration of 10 ng/μl in sterile distilled water along with competitor DNA (Porcine Hybloc, Applied Genetics Laboratories). Each probe combination contained a probe isolated from the distal p‐arm (labelled in FITC) and distal q‐arm (labelled in Texas Red) from a single chromosome. For acrocentric chromosomes, the most proximal sequence was isolated (for simplicity's sake, these were individually assigned with the chromosome number followed by the letter p in green type and the letter q in red type, as indicated in Fig.  S1).

The new device was based on the work of Knight *et al*. (1997) using a proprietary Chromoprobe Multiprobe^®^ System device manufactured by Cytocell Ltd. in the UK. Each probe combination (e.g. 1pq) was air dried onto a square of the device in the orientation indicated in Fig. S1. The corresponding glass slide was subdivided into 24 squares designed to align to the 24 squares on the device upon which chromosome suspensions were fixed.

### Fluorescence *in situ* hybridisation

Fixed metaphase preparations on the slide were dehydrated through an ethanol series (2 min each in 2× sodium saline citrate, 70%, 85% and 100% ethanol at room temperature). One microlitre of formamide‐based hybridisation buffer (Cytocell Hyb I) was pipetted onto each square of the device in order to resuspend the probes. The glass slide was aligned over the device (containing the rehydrated probes), pressed together and warmed on a 37 °C hotplate for 10 min. Probe and target DNA were subsequently denatured on a 75 °C hotplate for 5 min prior to hybridisation overnight in a dry hybridisation chamber in a 37 °C water bath. Following hybridisation, slides were washed (2 min in 0.4× sodium saline citrate at 72 °C; 30 s in 2× sodium saline citrate/0.05% Tween 20 at room temperature), then counterstained using DAPI in VECTASHIELD^®^ anti‐fade medium. Images were captured using an Olympus BX61 epifluorescence microscope with cooled CCD camera and SmartCapture (Digital Scientific UK) system. Each square of the slide was examined under the microscope, and a minimum of five metaphase spreads per square were captured. Where the probes in an individual square were shown to map to more than two chromosomes, the remaining squares were analysed to verify which of the other chromosomes were involved in the translocation. In the development phase, chromosome preparations from multiple animals were used to verify correct mapping of each BAC.

## Results and discussion

### Karyotype analysis

Karyotypes were successfully produced via a newly developed in‐house service for a total of 230 boars from different breeding populations with an average of 10 karyotypes created per boar. Four translocation carriers were identified by classical cytogenetics with no abnormalities identified in the remainder. The translocations were as follows t(1:2), t(7:10) (see Fig. 2), t(7:12) and t(13:15).

**Figure 1:**
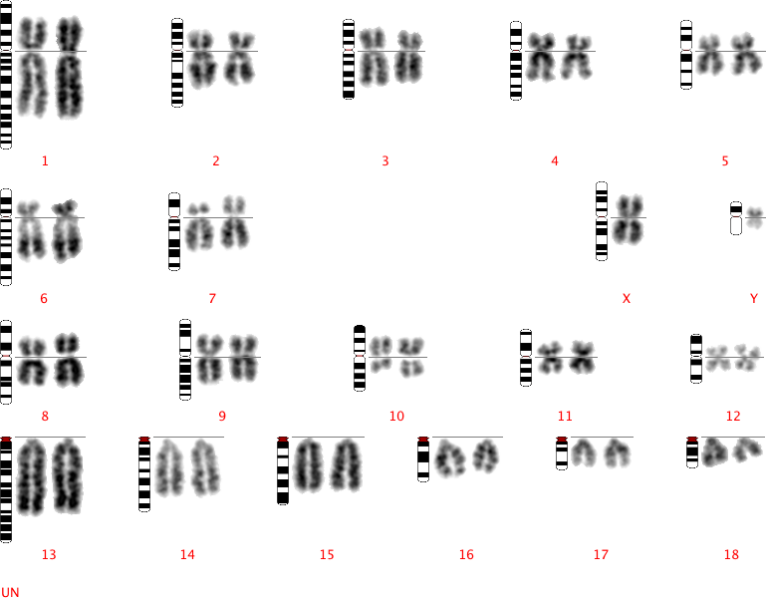
Standard DAPI‐banded karyotype of a boar carrying a 7:10 reciprocal translocation.

### Development of the Multiprobe device

**Figure 2:**
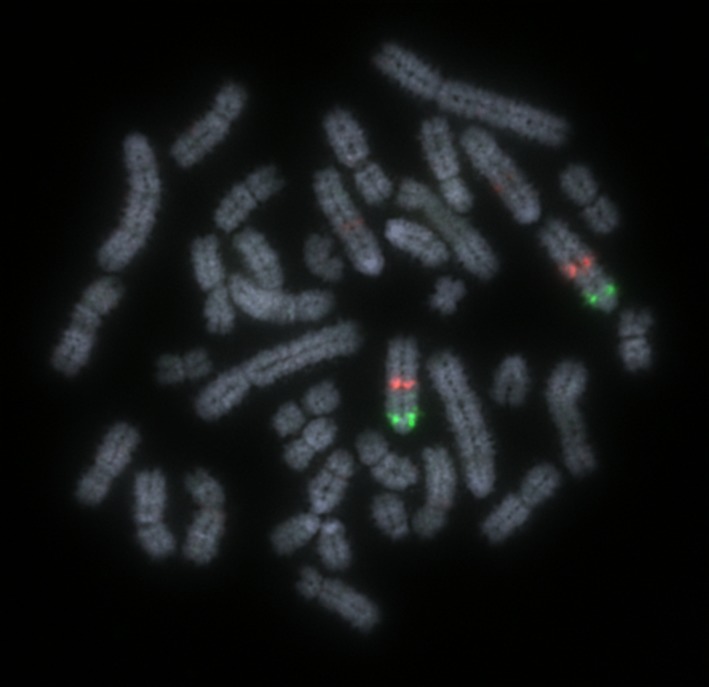
Clone ID PigE‐108N22 labelled in Texas Red, which should map to the distal end of SSC15 but appears halfway along this acrocentric chromosome. The FITC‐labelled probe mapped correctly. Scale bar 10 μm.

A total of 82 BACs were tested, of which ultimately 45 mapped correctly and 37 did not map as anticipated. All FITC‐labelled probes mapped to the expected locus at or near the p‐terminus of the chromosome with the exception of the BAC for chromosome 1p (PigE‐134L21), which actually mapped to chromosome 8), along with a BAC for chromosome 10p (PigE‐231H10), which mapped to chromosome 3, and three BACs originally assigned to chromosome 9p, which mapped to the centromeric region of chromosome 9. After the selection of alternative BACs, signals were observed at the appropriate end of the chromosome. Surprisingly, 32 of the 51 probes that were originally assigned to the q‐terminus of specific chromosomes mapped to a place in the genome other than that which was predicted. Of these, 24 clones (75%) mapped to the correct chromosome but not to the q‐terminus. An example is given in Fig. 2 for chromosome 15, and the full list given in Table 1.

**Table 1:** Incorrectly mapped porcine bacterial artificial chromosomes (BACs) and their assignment in the pig genome as revealed by fluorescence *in situ* hybridisation (FISH)

Chromosome	Arm	Clone name	FISH assignment	Same chromosome?
1	p	PigE‐134L21	8 p‐arm	No
1	q	CH242‐137C1	10 centromere	No
1	q	CH242‐35I10	Multiple	No
1	q	CH242‐83P21	7 centromere	No
2	q	CH242‐188K23	2 centromere	Yes
2	q	CH242‐230M23	2 centromere	Yes
2	q	CH242‐441A1	2 centromere	Yes
2	q	PigE‐117G14	2 p‐arm	Yes
3	q	CH242‐265K24	3 p‐arm	Yes
3	q	PigE‐221G14	3 p‐arm	Yes
3	q	PigE‐264D16	3 p‐arm	Yes
5	q	CH242‐133F9	5 p‐arm	Yes
5	q	CH242‐288F8	5 p‐arm	Yes
5	q	PigE‐127K14	5 p‐arm	Yes
5	q	PigE‐178M22	5 p‐arm	Yes
7	q	CH242‐272F22	7 centromere	Yes
7	q	CH242‐518F14	7 centromere	Yes
7	q	PigE‐208I10	3 q‐arm	No
7	q	PigE‐230H8	7 centromere	Yes
7	q	PigE‐75E21	7 mid q‐arm	Yes
9	p	CH242‐215O14	9 centromere	Yes
9	p	CH242‐44O5	9 centromere	Yes
9	p	CH242‐178L4	9 centromere	Yes
10	p	PigE‐231H10	3 p‐arm	No
10	q	CH242‐237D22	10 centromere	Yes
10	q	CH242‐36D16	10 q‐arm + extra signal on 1q	Yes
10	q	PigE‐60N24	1 centromere	No
11	q	PigE‐199B10	11 p‐arm	Yes
11	q	PigE‐232N19	11 p‐arm	Yes
15	q	PigE‐108N22	15 mid q‐arm	Yes
16	q	CH242‐4G9	16 p‐arm	Yes
16	q	PigE‐124C22	16 p‐arm	Yes
16	q	PigE‐173H6	16 p‐arm	Yes
17	q	PigE‐112L22	10 centromere	No
18	q	PigE‐141I21	6 p‐arm	No
X	q	CH242‐447L20	X p‐arm	Yes
X	q	PigE‐214O4	13 centromere	No

The results therefore indicated that probes assigned to the q‐arm were frequently incorrectly mapped, with the majority of probes mapping to the correct chromosome but the incorrect locus. Correctly mapping q‐arm probes were eventually assigned by choosing BACs (using an *in silico* approach) that were assigned to larger, fully mapped contigs closest to the q‐terminus.

**Figure 3:**
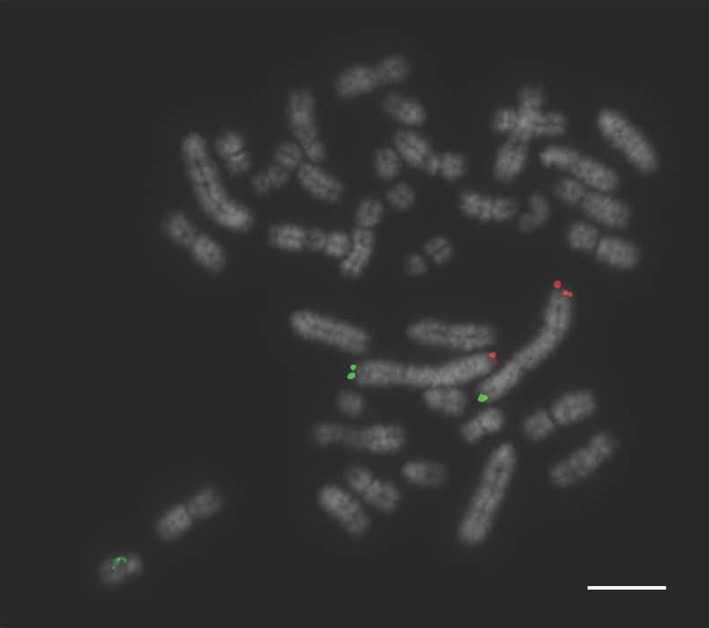
FISH image of correctly mapping bacterial artificial chromosome (BAC) clones for chromosome 1 tested on a chromosomally normal sample showing clear, punctate signals. Scale bar 10 μm.

**Figure 4:**
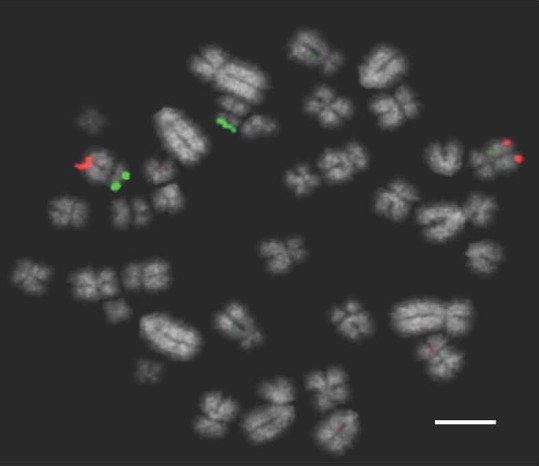
Labelled probes for *Sus scrofa* chromosome 7 (SSC7) illustrating a reciprocal translocation between SSC7 and SSC10. Scale bar 10 μm.

**Figure 5:**
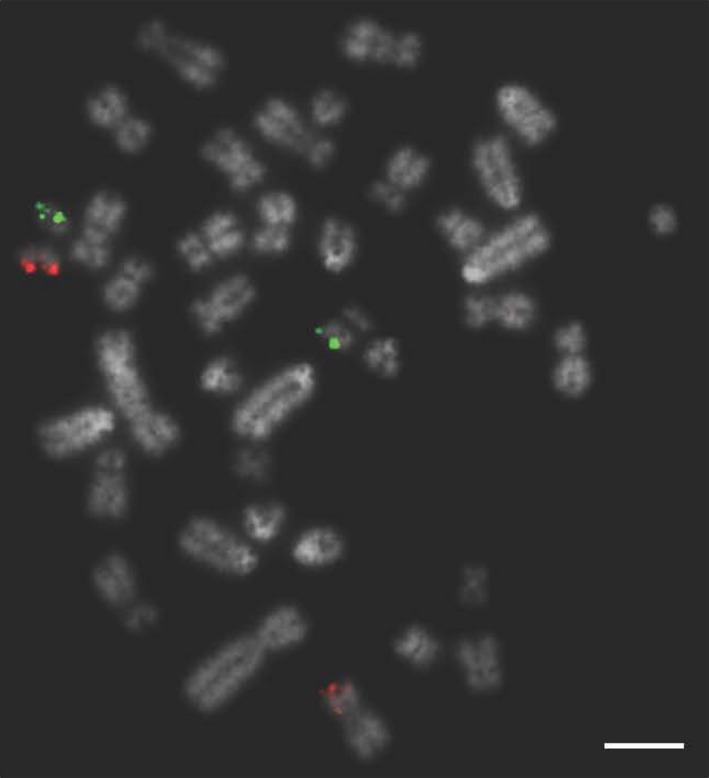
Labelled probes for *Sus scrofa* chromosome 10 (SSC10) illustrating a reciprocal translocation between SSC7 and SSC10. Scale bar 10 μm.

Ultimately, a device was developed and tested rigorously that gave bright, punctate signals (one green, one red) for each chromosome. Examples of the signals on chromosome 1 in a chromosomally normal preparation are given in Fig. 3. The newly developed Multiprobe strategy was applied to 21 chromosomally normal preparations and each translocation carrier in order to confirm the cytogenetic diagnosis. The device confirmed the diagnosis of the following translocations: t(1:2), t(7:10) (Figs. 4 & 5), t(7:12) and t(13:15). Moreover, no abnormalities were seen in the other preparations. A full list of subtelomeric BACs that give bright signals on the appropriate chromosome arms is shown in Table 2.

**Table 2:** Correctly mapping subtelomeric bacterial artificial chromosomes (BACs) for each porcine chromosome arm as revealed by fluorescence *in situ* hybridisation (FISH)

Chromosome	Arm	Clone name	Chromosome	Arm	Clone name
1	p	CH242‐248F13	10	q	CH242‐517L16
1	q	CH242‐151E10	11	p	PigE‐211E21
2	p	PigE‐8G19	11	q	CH242‐239O11
2	q	CH242‐294F6	12	p	PigE‐253K5
3	p	PigE‐168G22	12	q	PigE‐124G15
3	q	CH242‐315N8	13	P	PigE‐197C11
4	p	PigE‐131J18	13	q	PigE‐179J15
4	q	PigE‐85G21	14	p	PigE‐137C12
5	p	PigE‐74P10	14	q	PigE‐167E18
5	q	CH242‐63B20	15	p	PigE‐90C11
6	p	PigE‐238J17	15	q	CH242‐170N3
6	q	CH242‐510F2	16	p	PigE‐149F10
7	p	PigE‐52L22	16	q	CH242‐42L16
7	q	CH242‐103I13	17	p	CH242‐70L7
8	p	PigE‐2N1	17	q	CH242‐243H19
8	q	PigE‐118B21	18	p	PigE‐253N22
9	p	CH242‐65G4	18	q	PigE‐202I11
9	q	CH242‐411M8	X	p	CH242‐19N1
10	p	CH242‐451I23	X	q	CH242‐305A15

**Figure 6:**
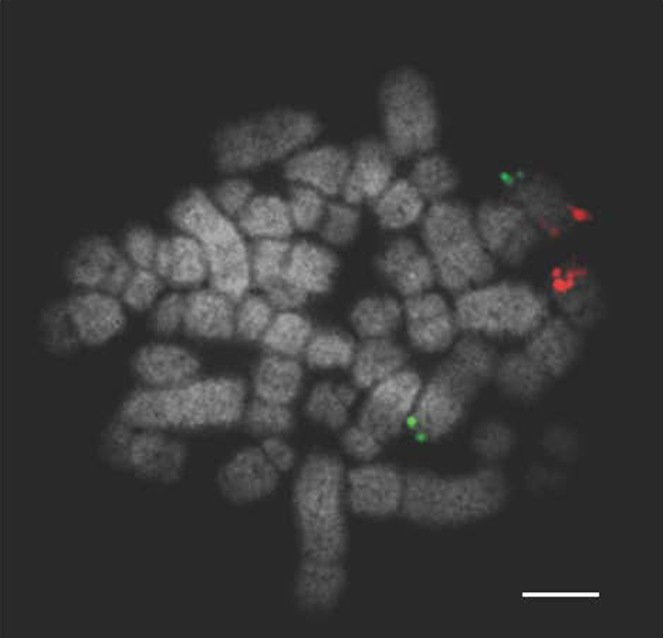
Bacterial artificial chromosome (BAC) clones for *Sus scrofa* chromosome 5 (SSC5; (p‐arm labelled in FITC and q‐arm labelled in Texas Red) showing a translocation between SSC 5 and 6. Despite the suboptimal chromosome preparation the translocation is clearly visible. Scale bar 10 μm.

**Figure 7:**
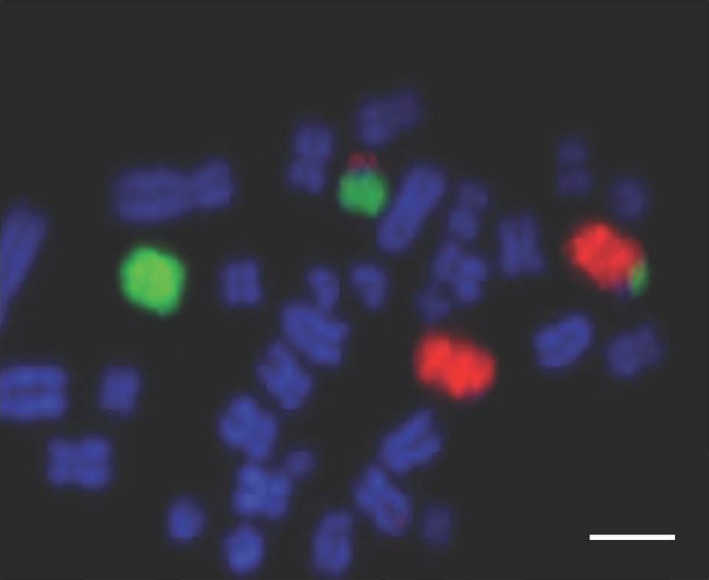
Chromosome paints for *Sus scrofa chromosome* 5 (SSC5) FITC) and SSC6 (Texas Red) illustrating the cryptic translocation that had been previously undetectable from the karyotype. Scale bar 10 μm.

An additional boar that had previously been diagnosed as karyotypically normal was retested using the Multiprobe device, which revealed a chromosome translocation between chromosomes 5 and 6 that was missed by classical karyotyping (Fig. 6). Further analysis with chromosome painting for porcine chromosomes 5 and 6 on this boar revealed a cryptic translocation with the distal portions of the two chromosomes exchanged (Fig. 7). Karyotyping was limited by sub‐optimal quality of the original chromosome preparation, however results produced using the FISH approach clearly identified the translocation despite the poor preparation and the small size of the translocation.

Results of this study provide proof of principle of an approach that can be used successfully to diagnose chromosomal translocations that directly impact fertility in pigs at a resolution previously difficult to achieve by standard karyotyping. There are three advantages of using this approach over classical karyotyping. The first is that it detects more cryptic translocations than standard karyotyping otherwise would. The boar indicated in this study is an example. Indeed, the fact that a previously undetected cryptic translocation was identified would suggest that the actual number of translocations in the boar breeding population might in fact be significantly higher than previously reported. It is possible that these karyotypically cryptic and unreported translocations are seen more frequently than expected but that the routine use of multiple inseminations per sow may be diluting the effect on the farrowing rates. The boar with a cryptic translocation in this study had a significantly reduced farrowing rate and interestingly also had a significantly lower ‘born dead’ rate, suggesting that the translocation in this case results in early embryonic loss. It would appear that the production of unbalanced gametes caused by the translocation in question results in embryos that are not compatible with early life, causing early embryo mortality in a pattern that is also seen in humans (Tempest & Simpson 2010). In humans, reciprocal translocations arise more frequently *de novo* rather than from being inherited from a carrier parent (Tempest & Simpson 2010). It would therefore be reasonable to suggest that the same pattern of familial inheritance applies to pigs and other animals. The *de novo* nature of these translocations supports the theory that all boars awaiting service should be screened chromosomally to reduce the risk of using a hypoprolific animal for breeding purposes. In fact, despite over 130 reciprocal translocations being reported in the literature, to date this is the first reported translocation to have occurred between chromosomes 5 and 6, suggesting that this fits that category (Rothschild & Ruvinsky 2011). Secondly, as in this case, when preparations are sub‐optimal, this approach provides necessary ‘back‐up’ to ensure accurate diagnosis. That is, provided FISH signals are clear enough, confident diagnosis can be made on a single metaphase, regardless of the length of the chromosomes.

The final issue is that the device permits analysis by individuals who are less well trained in karyotype analysis. Twenty years of experience of teaching students to karyotype human and pig karyotypes (Gibbons *et al*. 2003; Morris *et al*. 2007) has demonstrated that the technical skills required to produce a karyotype reliably can be variable between individuals and that animal‐specific expertise is invaluable. Indeed, although several laboratories have pioneered animal cytogenetics for the purposes of AI boar (and bull) screening, there are fewer now than in previous decades despite the need to continue screening in this manner. Nonetheless, it should be made clear that specialist cytogenetic skills are still required to make chromosome preparations reliably in the lab and to perform overall analyses. The scheme developed here should therefore be considered an adjunct to classical cytogenetics, not a replacement for it.

A second outcome of this study was the revelation that a large number of BACs isolated from the swine genome assembly mapped incorrectly. That is, those that were predicted to map to the q‐terminus of a particular chromosome mapped elsewhere on the same chromosome. In many ways, this contradicts our previous results in which 100% of the BACs mapped to the predicted chromosomal location (Groenen *et al*. 2012). The high level of mapping errors found in this study led to further investigation of the clone placement with members of the Swine Genome Sequencing Consortium. It became evident that the problem was the result of some errors in the way in which parts of the draft pig genome sequence were assembled. Specifically, analysis of the BAC sequences revealed that the high error rate was due to misplacement of some of the smaller fingerprint contigs within which the BAC was located. These small fingerprint contigs did not have full sequence and orientation data when the genome was assembled, and it appears that these small poorly mapped contigs were added to the end of the list of contigs for the relevant chromosomes. This resulted in the sequences from the BACs in these poorly mapped contigs being randomly added to the end of the relevant chromosomes, which explains why the error rate was particularly high among BACs chosen to map to the subtelomeric q‐arm region.

The genome assembly errors found throughout the course of this project highlight the need for caution when choosing BACs for this purpose. In other words, the porcine genome assembly still appears to have assembly flaws, despite being initially considered to be one of the best assembled. These assembly errors are particularly apparent when looking at structural rearrangements and should be taken into consideration when planning future FISH mapping exercises, both for BACs in the pig genome and when investigating the genomes of other animal species (e.g. cattle, sheep). The errors highlighted in this paper have been passed on to the Swine Genome Sequencing Consortium, and the results will be incorporated in an improved pig genome assembly due to be released in 2016. With the rapid expansion in the number of newly sequenced animal genomes being published, along with corresponding BAC libraries for many, the possibility of assembly errors should be an important consideration for future similar studies.

Now that a full set of porcine subtelomeric probes has been identified and applied in the manner described, screening efficiency can be improved by allowing the analysis of the full chromosomal complement on one slide. Given the nature of translocations and their impact on fertility in pigs, the simple, rapid identification of (cryptic or otherwise) translocations will facilitate the detection and subsequent removal of affected animals from the breeding population at an early stage. This has the potential to lead to long‐term improved productivity and delivering meat products in a more cost‐effective and environmentally friendly way to a growing population. The widespread use of artificial insemination and the large market for superior boar semen being sold to both small‐ and large‐scale pig breeding operations suggests that improvements in productivity impact not just the large commercial breeders but also the smaller farmers for whom reduced wastage may be more critical.

Finally, the application of these subtelomeric FISH probes for translocation screening is not necessarily limited to screening for translocations in pigs. Artificial insemination is also widely used in cattle breeding, with a high premium placed on bull semen of superior genetic merit. With sufficient alterations (i.e. incorporating cattle subtelomeric BACs), the device could be adapted to this and other species. In addition, the increasingly widespread use of embryo transfers in cattle would suggest that the cow and the bull should both be screened for chromosomal translocations. In fact, the cattle karyotype is more difficult to analyse reliably because of a diploid number of 60, largely made up of similar‐sized acrocentric chromosomes. The cattle karyotype therefore lends itself to the use of a FISH‐based screening approach such as is described here, as does the largely acrocentric sheep karyotype (2n = 54). Lessons regarding genome assembly learnt from this exercise would suggest that a cautionary approach be taken when identifying BACs for this purpose and that a combined *in silico* and experimental approach is crucial in the development of similar tools.

## Conclusions

The FISH‐based translocation screening technique developed in this study is a powerful and reliable approach to translocation screening with great potential to be adapted to other species. Development of this method also resulted in the identification of errors in the pig genome assembly, the resolution of which will be of benefit to the pig genome community.

## Competing interests statement

Martin Lawrie, Richard Frodsham and Gothami Fonseka are employees of Cytocell Ltd; Grant Walling is an employee of JSR Genetics. Both companies could, in theory, profit from publication of this manuscript. Rebecca O'Connor, Darren Griffin and Alan Archibald declare no conflict of interests except in that they have collaborated with these companies to generate successful co‐funded grant applications. At time of writing, the device described above is now a commercial product marketed by Cytocell.

All institutional and national guidelines for the care and use of laboratory animals were followed. ALA was supported by BBSRC (BB/J004235/1, BB/I025328/1, BB/M011461/1).

## Supporting information


**Figure S1** Multiprobe device layout of labelled bacterial artificial chromosome (BAC) clones by chromosome with a Texas Red labelled probe and FITC‐labelled probe for each chromosome air dried onto the same square.
