# Supplementary material for: Isolation of subtelomeric sequences of porcine chromosomes for translocation screening reveals errors in the pig genome assembly
Source: Anim Genet. 2017 May 12;48(4):395–403. doi: 10.1111/age.12548 (PMC5518436; doi:10.1111/age.12548)

**Figure S1** Multiprobe device layout of labelled bacterial artificial chromosome (BAC) clones by chromosome with a Texas Red labelled probe and FITC-labelled probe for each chromosome air dried onto the same square.


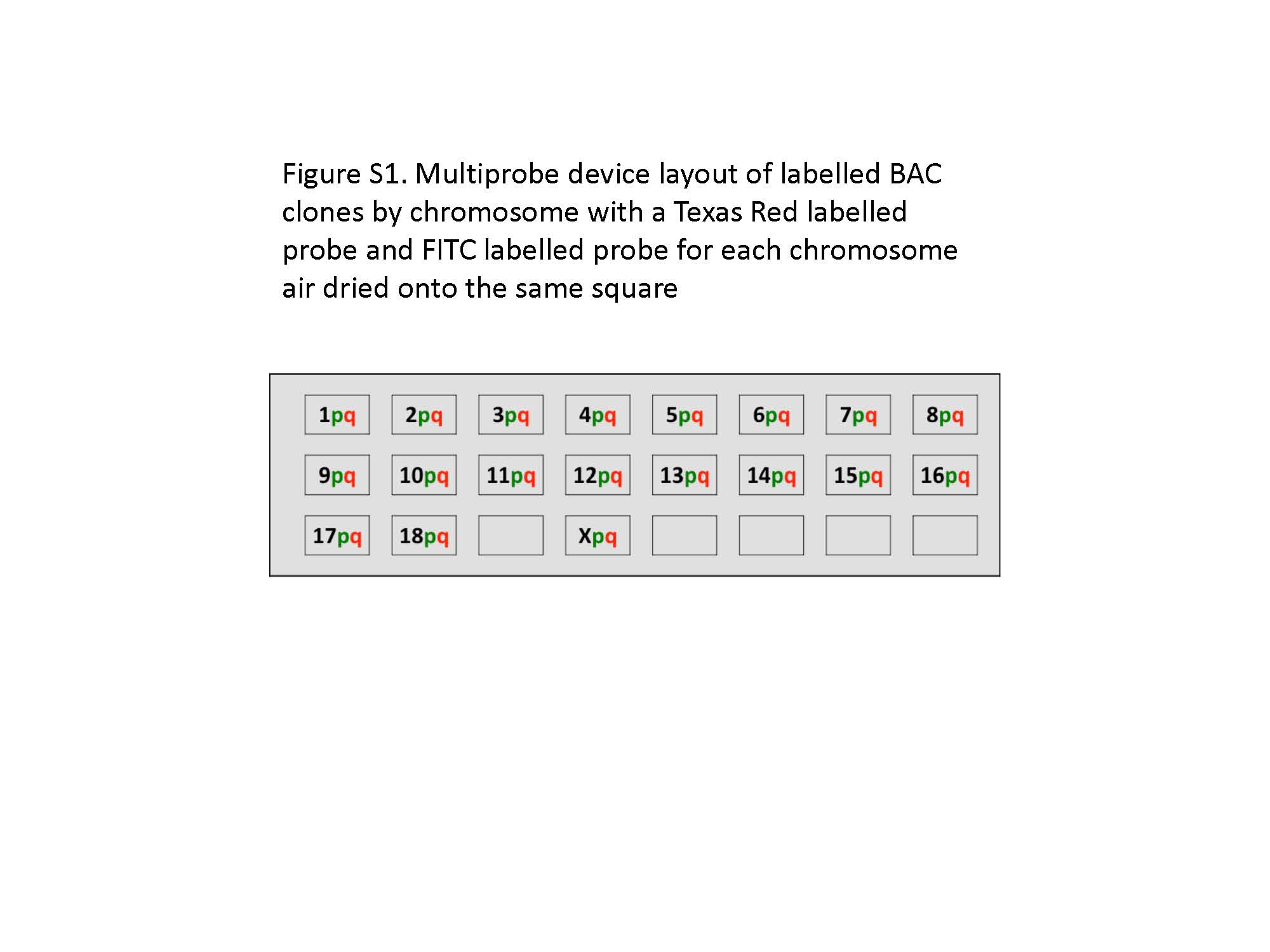

Supplement: Supplementary file 1 — Figure S1 Multiprobe device layout of labelled bacterial artificial chromosome (BAC) clones by chromosome with a Texas Red labelled probe and FITC‐labelled probe for each chromosome air dried onto the same square. [file AGE-48-395-s001.docx]
